# Staff satisfaction with the use of bedside electronic transfusion checks at three hospitals in London

**DOI:** 10.1111/vox.70282

**Published:** 2026-05-06

**Authors:** Florence Oyekan, Montasir Ahmed, Catherine Booth, Louise Bowles, Ollie Djurdjevic, Yan Feng, Kirsty Hancock, Suzanne Makki, Helinor McAleese, Josephine McCullagh, Khin Mon, Michael F. Murphy, Simon J. Stanworth, Nathan Proudlove, Laura Green

**Affiliations:** ^1^ Blizard Institute, Barts and the London School of Medicine and Dentistry Queen Mary University of London London UK; ^2^ Barts Health NHS Trust London UK; ^3^ Wolfson Institute of Population Health Queen Mary University of London London UK; ^4^ NHS Blood and Transplant London UK; ^5^ Blood & Transplant Research Unit, NDCLS, Radcliffe Department of Medicine University of Oxford Oxford UK; ^6^ Oxford University Hospitals NHS Trust Oxford UK; ^7^ Allliance Manchester Business School University of Manchester Manchester UK

**Keywords:** bedside electronic transfusion checks (BETC), blood administration, personal digital assistant (PDA), positive patient identification (PPID), sample labelling, staff satisfaction and perspective, survey

## Abstract

**Background and Objectives:**

Bedside transfusion errors, especially positive patient identification (PPID), are a risk to patient safety. Bedside electronic transfusion checks (BETC), using barcode‐enabled personal digital assistants (PDAs), are recommended to improve safety and efficiency. This study assessed staff satisfaction with BETC versus manual transfusion checks in three large London hospitals. The surveys aimed to compare clinical staff satisfaction with BETC versus the manual system.

**Materials and Methods:**

A cross‐sectional survey was conducted immediately after training and 6 months after routine BETC use. The initial (21 questions) and follow‐up (15 questions) surveys assessed usability, accuracy, workflow efficiency and patient care impact. Responses were collected via Microsoft Forms and analysed using descriptive statistics and logistic regression, adjusting for job role, experience and hospitals.

**Results:**

A total of 2085 staff completed the initial survey (55% response) and 514 the follow‐up (13%), predominantly nurses (75%). For group and screen (G&S) labelling, ratings of ‘ease of use’ and ‘accuracy’ improved significantly between surveys, while perceived impact on reducing mislabelling remained consistently high (96.2% vs. 94.7%). Compared with manual checks, BETC was rated significantly by clinicians for ease of use (89% → 94%) and accuracy (89% → 95%; both *p* < 0.001). Improvements were also observed for the time saved by clinical staff (75% → 89%), patient care (77% → 89%), fewer nurses required (79% → 91%) and traceability (80% → 87%), all statistically significant (*p* < 0.001).

**Conclusion:**

BETC was associated with significantly greater clinical staff satisfaction than manual transfusion checks, providing large‐scale evidence for their adoption to enhance transfusion safety, efficiency and staff experience.

**Highlights**

- Bedside electronic transfusion checks (BETC) was associated with significantly greater clinician satisfaction than manual transfusion checks.
- Respondents agreed that use of the personal digital assistant (PDA) system would reduce the rejection rate of mislabelled samples.
- Clinical staff found the bedside transfusion checks easy to use for sample labelling and blood administration.

## INTRODUCTION

Errors relating to positive patient identification (PPID) at the bedside are the most common reasons for the wrong blood component for transfusion being given to the wrong patients [1]. To mitigate these risks, UK national guidelines recommend multiple additional safety measures when using manual bedside systems. These include collecting two separate group and screen (G&S) samples, adopting a zero tolerance policy on mislabelled G&S specimens in the laboratory and mandating a two‐person PPID check prior to every blood unit administration [2, 3, 4].

In the United Kingdom, electronic blood management systems [5] have been recommended to improve transfusion safety and efficiency. For the bedside steps, such systems use a barcode scanning technology that enables an automated electronic check for G&S sample labelling and blood component administration against the patient's identification details at the bedside and in the Laboratory Information Management System (LIMS). Despite these recommendations, the uptake of bedside electronic transfusion checks (BETC) remains low in the country [6], with a recent audit [7] showing that only 36% of National Health Service (NHS) Trusts in England (91.4% of eligible trusts participated in the audit) responding to the survey have an electronic bedside system for pre‐transfusion checks [8].

The reasons for poor uptake of electronic systems are unclear. Healthcare professionals, particularly nurses, play a central role in transfusion pathways, including patient identification and blood administration [9]. As transfusion procedures become increasingly digitized, the impact of expanded adoption of electronic capabilities on staff workflow and perceptions remains insufficiently explored. The users' attitude towards these systems is of importance in optimizing compliance with their uptake and the overall service provision for patients [10, 11, 12]. Assessing healthcare professionals' perceptions of newly implemented digital technologies is critical to ensuring their successful integration, ongoing use and impact on patient safety—particularly in high‐risk clinical processes such as blood transfusion [13, 14, 15]. Nurses and clinical support staff, who are directly involved in bedside transfusion checks, play a pivotal role in ensuring the safety and accuracy of these processes [9, 11]. Their acceptance and satisfaction with tools such as BETC significantly influence compliance, workflow efficiency and overall quality of care [10, 15].

The aim of this study was to assess staff satisfaction with the use of BETC during the implementation of BETC across four hospitals in London and compare the findings to the previous manual system.

## MATERIALS AND METHODS

Barts Health NHS Trust is comprised of four hospitals, all of which have a dedicated transfusion laboratory. Between them they administer around 65,000 units of blood components per year, and around 6500 staff are involved in blood transfusion procedures across the four hospitals in 193 wards. The Trust provides some of the busiest trauma, cardiac and haemoglobinopathy services in the United Kingdom and has significant medical, surgical and paediatric services.

In April 2022, the Trust made the decision to replace the manual bedside checks for sample labelling for blood compatibility testing and blood administration with bedside electronic transfusion devices. The devices consisted of a portable handheld scanning device (or personal digital assistant [PDA]) and a mobile printer, supplied by Haemonetics©. Prior to the devices being used in clinical wards, the assigned transfusion practitioners trained the staff mostly in groups of three or more, with training sessions taking on average 60 min. All staff underwent face‐to‐face training, except two who attended the training online.

### Surveys

The project received research ethics approval (no. 22/NW/0138) and Health Research Authority approval for data collection (IRAS no. 311676), and an approved study protocol [16]. To compare users' satisfaction with the new system, a similar anonymized survey was distributed to all clinical staff who underwent training for BETC at two time points: (1) immediately after training, and (2) at 6 months after the training (survey questions are given in Appendix A). The reason for repeating the survey was to allow users more time to familiarize themselves [17] with the devices and to determine whether their satisfaction with BETC improved over time.

Anonymized electronic surveys were designed using the online Microsoft Forms tool and structured into a mixture of open‐ended and closed‐ended questions. The first survey included 21 questions, while the second survey comprised 15 questions. The survey was split into three main sections: *Section 1* collected general information about individuals (e.g., hospital where they are practicing, job role, number of years working in the role). This section also included additional contextual questions that—while not directly related to clinicians' views on the use of the PDA system—provided useful implementation context. These included the date of training received, the ward/department they were working in, their clinical speciality, how the training was delivered (e.g., in person or virtual), whether the session was a one‐off or a refresher, suggestions and feedback on how to improve the service and the duration of the training. *Section 2* focused on comparisons of manual versus BETC for the G&S blood sample labelling, and *Section 3* focused on comparisons of pre‐transfusion administration checks between manual versus BETC. For Sections 2 and 3, we posed questions towards the ‘ease of use’; ‘speed’ and ‘accuracy’ of performing tasks and ‘outcome’ of the tasks (e.g., for G&S sample labelling, the outcome was rejection rate and for pre‐administration checks it was improvement in patient care, healthcare time and traceability).

Users were asked to complete the first survey immediately following the training session (i.e., face to face) or online. The second survey requests were sent via email, with clinicians invited to participate after 6 months experience with the use of BETC. The survey was piloted by 47 staff prior to being finalized, and the pilot results were pooled with the main results, as there were no significant changes made to the survey following the pilot.

To distinguish responses by the user group, the questionnaire included conditional questions that enabled the user to complete only questions relevant to their role and responsibility. No financial incentive was offered for the survey. The survey was voluntary and was distributed to all staff trained to use BETC. Responses were accepted between May 2023 and April 2025. A single response was submitted per staff member.

### Analysis

Descriptive statistics [18, 19, 20, 21] (frequencies and percentages) were used to summarize clinical staff responses from the two surveys, including demographics and outcomes. As the surveys were anonymized, individual responses could not be linked between surveys; therefore, no statistical tests of association were conducted.

To examine factors associated with clinical staff satisfaction, we fitted logistic regression models, which are well established in clinical research for estimating the probability of categorical outcomes, while adjusting for covariates [22, 23, 24]. To assess changes over time, we compared responses from two surveys with the key independent variables being the surveys (0 = immediately after training; 1 = 6 months after training). Covariates included years of experience, job role and hospital site. For ordinal outcomes, rated on scales as ‘excellent’ to ‘poor’, we present responses based on positive versus negative perceptions and apply binary logistic regression. The primary survey outcomes, such as ‘ease of use’ and ‘accuracy of performing steps’, were measured on ordered categorical scales (‘excellent’, ‘very good’, ‘good’, ‘fair’, ‘poor*’*). To simplify interpretation and evaluate overall positive versus negative perceptions, responses of ‘excellent’, ‘very good’ or ‘good’ were coded as 1 (positive), while ‘fair’ and ‘poor’ were coded as 0 (negative). Outcomes related to ‘patient care’, ‘healthcare professional time’, ‘traceability of blood components’ and ‘staffing requirements’ were measured on agreement scales (‘strongly agree’, ‘agree’, ‘neither agree nor disagree’, ‘disagree’, ‘strongly disagree’). Here, ‘strongly agree’ and ‘agree’ were coded as 1 (positive), while ‘disagree’, ‘strongly disagree’, and ‘neither agree nor disagree’ were coded as 0 (negative). Binary logistic regression (logit) models were then used to estimate the probability of a positive response, adjusting for relevant covariates.

To improve interpretability, we report adjusted predicted probabilities, using the margins command [25, 26] using Stata (version 18.0, StataCorp LLC), which provides an intuitive summary of model results by estimating the average probability of each outcome category at different levels of the main explanatory variable while holding other covariates constant [27]. This approach facilitates a clearer understanding of results, particularly when dealing with models that include multiple categorical predictors. We analysed the data and figures using Excel, STATA and Python software.

## RESULTS

### Demographics

The surveys were sent to 3825 staff: of these, we received responses from 2085 (55%) staff for the first survey and 514 (13%) responses for the second survey. Responses to the survey were received from a wide range of diverse job roles, with most of the staff in both surveys being nurses (75%). The distribution of staff job roles was similar for both surveys. Further details on staff demographics, their years of training experience and transfusion training are provided in Table 1.

**TABLE 1:** Demographics of health professionals' respondents.

Characteristics	First survey	Second survey
	*N* = 2085, *n* (%)	*N* = 514, *n* (%)
Job category		
Nurse	1631 (78.2)	384 (74.7)
Doctor	173 (8.3)	36 (7.0)
Support group	156 (7.5)	49 (9.5)
Theatre group	116 (5.6)	44 (8.6)
Others	9 (0.4)	1 (0.2)
Job experience		
<2 years	624 (29.9)	78 (15.2)
2–3 years	263 (12.6)	49 (9.5)
3–4 years	149 (7.1)	68 (13.2)
4–5 years	130 (6.2)	98 (19.1)
5+ years	918 (44.0)	221 (43.0)
Hospital		
Hospital 1	531 (25.5)	194 (37.7)
Hospital 2	651 (31.2)	203 (39.5)
Hospital 3	902 (43.3)	117 (22.8)
Trained to perform G&S sample labelling		
No	257 (12.3)	45 (8.7)
Yes	1827 (87.6)	469 (91.2)
Trained to perform blood administration		
No	285 (15.6)	71 (15.1)
Yes	1542 (84.4)	398 (84.9)

Abbreviation: G&S, group and screen.

The two surveys showed broadly similar clinician demographics. Respondents primarily worked in frontline acute services, including Accident and Emergency (A&E), critical care and peri‐operative areas (anaesthetics, theatres, surgery), with strong representation from women's and children's services (maternity, obstetrics, neonates/paediatrics). A wide range of other specialties also contributed, including renal, cardiology, respiratory, oncology, gastroenterology and haematology.

### G&S blood sample labelling

Table 2 presents health professionals' perceptions regarding the use of BETC system for G&S blood sample labelling between the first (*N* = 2085) and second (*N* = 514) surveys. For both questions (‘ease of use’ and ‘accuracy’ of performing labelling steps), the proportion of respondents rating the system as ‘excellent’ and ‘very good’ increased significantly between the two surveys, while the ratings of ‘good’ and ‘fair’ decreased significantly, indicating greater user satisfaction with experience and usability over time. Finally, the perceived impact on mislabelling remained consistently high across both surveys. Most respondents in the first (96.2%) and second survey (94.7%) believed that using the PDA system would reduce the rejection rate of mislabelled samples.

**TABLE 2:** Level of satisfaction from health professionals on the use of bedside electronic transfusion checks for blood sample labelling.

	First survey	Second survey
	*N* = 2085, *n* (%)	*N* = 514, *n* (%)
Ease of use		
Excellent	512 (28.0)	192 (40.9)
Very good	494 (27.1)	199 (42.4)
Good	615 (33.7)	53 (11.3)
Fair	187 (10.2)	16 (3.4)
Poor	18 (0.9)	9 (1.9)
Accuracy of performing steps		
Excellent	563 (30.8)	206 (43.9)
Very good	489 (26.8)	192 (40.9)
Good	578 (31.6)	48 (10.2)
Fair	184 (10.1)	18 (3.8)
Poor	12 (0.6)	5 (1.0)
Will PDA use reduce rejection rate of mislabelled samples?		
Yes	1758 (96.2)	444 (94.7)
No	69 (3.8)	25 (5.3)

Abbreviation: PDA, personal digital assistant.

In Survey 2, 68.1% of respondents found the PDA quicker than the manual method for G&S blood labelling. In Survey 1, responses showed manual checks received mixed ratings (29.7% slow, 7.7% ‘very slow’, 24.7% ‘fast’, 15.6% ‘very fast’), whereas electronic checks were more favourable, with 74.9% rating them as ‘fast’ or ‘very fast’ with only 7.6% reporting electronic checks as slow.

### Blood administration

Table 3 shows the results of both surveys on health professionals' perceptions regarding the use of the PDA system for blood administration checks. Between the first and the second survey, there was a significant increase in users' perception for (a) rating the system as ‘excellent’ or ‘very good’ for ‘ease of use’ and ‘accuracy of performing steps’ and (b) improving ‘healthcare professionals' time’, ‘patient care’ ‘fewer nurses required for checks' and ‘traceability’. Participants were also asked to rate their overall satisfaction with using a PDA for pre‐transfusion checks on a scale from 0 (not at all satisfied) to 10 (extremely satisfied).

**TABLE 3:** Level of satisfaction from health professionals on the use of bedside electronic transfusion checks for blood administration.

	First survey	Second survey
	*N* = 2085, *n* (%)	*N* = 514, *n* (%)
Ease of use		
Excellent	517 (28.7)	181 (40.8)
Very good	500 (27.8)	175 (39.5)
Good	599 (33.3)	59 (13.3)
Fair	162 (9.0)	17 (3.8)
Poor	20 (1.1)	11 (2.8)
Accuracy of performing steps		
Excellent	540 (30.0)	197 (44.4)
Very good	512 (28.5)	165 (37.3)
Good	583 (32.4)	57 (12.9)
Fair	145 (8.1)	14 (3.1)
Poor	18 (1.0)	10 (2.3)
Improve healthcare professionals' time		
Strongly agree	649 (36.1)	231 (52.1)
Agree	696 (38.7)	159 (35.9)
Neither agree nor disagree	230 (12.8)	26 (5.9)
Disagree	65 (3.6)	9 (2.0)
Strongly disagree	158 (8.8)	18 (4.1)
Improve patient care		
Strongly agree	656 (36.5)	220 (49.7)
Agree	734 (40.8)	172 (38.8)
Neither agree nor disagree	222 (12.3)	23 (5.2)
Disagree	25 (1.4)	12 (2.7)
Strongly disagree	161 (8.9)	16 (3.6)
Fewer nurses are required for checks		
Strongly agree	736 (40.9)	279 (62.9)
Agree	688 (38.2)	123 (27.7)
Neither agree nor disagree	182 (10.1)	20 (4.5)
Disagree	30 (1.7)	6 (1.3)
Strongly disagree	162 (9.0)	15 (3.4)
Improve traceability of blood components		
Strongly agree	761 (42.3)	209 (47.9)
Agree	689 (38.3)	174 (39.3)
Neither agree nor disagree	158 (8.8)	37 (8.3)
Disagree	21 (1.1)	13 (2.9)
Strongly disagree	169 (9.4)	10 (2.3)
Overall satisfaction level with PDA use (scale 0–10)		
Low (0–3)	8 (0.4)	6 (1.4)
Moderate (4–7)	232 (12.9)	67 (15.2)
High (8–10)	1557 (86.6)	367 (83.4)

Abbreviation: PDA, personal digital assistant.

Overall satisfaction with the pre‐transfusion check was high across both groups, with most respondents rating the system between 8 and 10 (86% in the first survey and 83% in the second) and a smaller proportion rating it as moderate (scores 4–7) or as low (scores 0–3). Among respondents, missing data for G&S were 12.4% in Survey 1 and 8.8% in Survey 2, while missing data for blood administration were 13.8% in both surveys.

### Adjusted predicted probabilities of clinician feedback

**FIGURE 1:**
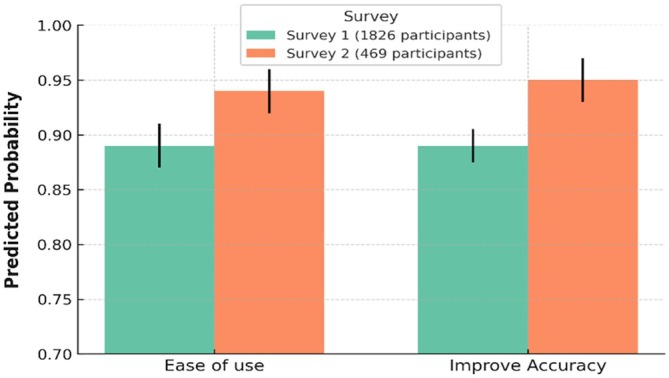
Adjusted predicted probabilities of satisfaction from health professionals on the use of bedside electronic transfusion checks (BETC) for blood sample labelling. The figure shows adjusted predicted probabilities for clinicians' reported ease of use and improved accuracy when performing blood sample labelling tasks before and after BETC implementation. The bar chart illustrates these adjusted predicted probabilities, with vertical black lines indicating 95% confidence intervals for each prediction.

Clinical staff rated BETC significantly higher than the manual checks (Figure 1) regarding ‘ease of use’ and ‘accuracy of performing steps’. The predicted probability for ‘ease of use’ increased from 89% (95% confidence interval [CI]: 0.88–0.91) in Survey 1 to 94% (95% CI: 0.92–0.96) in Survey 2. Similarly, the probability of reporting ‘improved accuracy’ rose from 89% (95% CI: 0.88–0.91) to 95% (95% CI: 0.93–0.97). All differences were statistically significant (*p* < 0.001).

**FIGURE 2:**
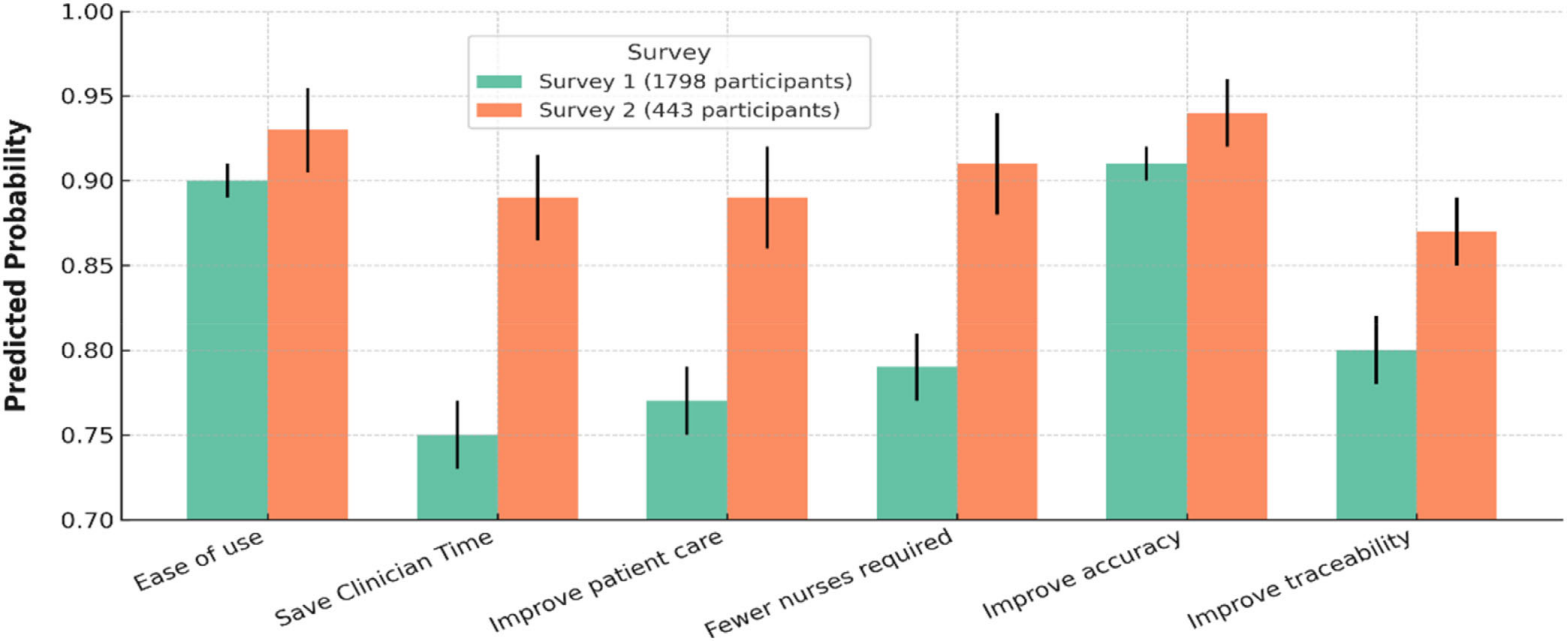
Predicted probabilities of satisfaction from health professionals on the use of bedside electronic transfusion checks (BETC) for blood administration. The figure presents adjusted predicted probabilities for clinician‐reported outcomes before and after the implementation of BETC. The bar chart displays adjusted predicted probabilities, with vertical black lines representing 95% confidence intervals for each predicted probability. Across all six outcomes measured, clinicians rated BETC more favourably than the manual method, with higher predicted probabilities observed in Survey 2 compared with Survey 1 (*p* < 0.001 for all comparisons).

Clinical staff consistently rated BETC more favourably than manual checks across all six outcomes (Figure 2). Predicted probabilities increased significantly from Survey 1 to Survey 2. Ease of use improved from 90% (95% CI: 0.89–0.91) to 93% (95% CI: 0.91–0.96), clinical staff time savings from 75% (95% CI: 0.73–0.77) to 89% (95% CI: 0.86–0.91) and patient care from 77% (95% CI: 0.75–0.79) to 89% (95% CI: 0.86–0.92). Similarly, ‘fewer nurses required’ increased from 79% (95% CI: 0.77–0.81) to 91% (95% CI: 0.88–0.94), ‘improved accuracy for performing steps’ from 91% (95% CI: 0.90–0.92) to 94% (95% CI: 0.92–0.96) and ‘traceability’ from 80% (95% CI: 0.78–0.82) to 87% (95% CI: 0.92–0.96). All differences were statistically significant (*p* < 0.001).

## DISCUSSION

Alongside the implementation of BETC, we surveyed staff to compare the new system to manual checks. Our findings offer a critical user‐centred perspective that complements existing performance metrics and highlights the value of integrating end‐user feedback into digital health evaluations. Participants reported more favourable perceptions of the digital system compared to manual checks, with significant improvements observed over time across all assessed domains, indicating that user familiarity is a critical determinant of system acceptance.

Previous studies on electronic blood transfusion systems have predominantly focused on improving workflow efficiency, process adherence and patient safety, including reductions in transfusion errors and turnaround times [5, 6]. These evaluations typically emphasized outcomes such as barcode scanning compliance, system integration and reduction of mislabelling or wrong blood in tube events [1, 28]. There is a lack of published evidence exploring staff satisfaction or frontline user perceptions of BETC systems, particularly in large multi‐hospital implementations. Understanding staff experience is essential, as successful adoption of such technologies depends not only on clinical outcomes but also on how end‐users perceive their usability, impact on workflow and value to patient care [15].

To maximize response consistency, surveys were administered immediately post training and repeated after a period of routine use. This two‐stage design enabled us to capture both the initial and sustained user experiences. The findings offer practical implications for the implementation of similar technologies within other NHS Trusts and international healthcare settings. Response rates declined from 55% to 13%, likely reflecting the difference between in‐person versus remote survey administration modalities. The first survey's response rate was similar to that of previous studies [7]; the second was lower despite engagement efforts [29], likely due to staff workload and time constraints [16, 30], especially among nurses (75%). Over 500 responses with consistent demographics allowed valid comparison.

BETC consistently received high satisfaction across usability, accuracy, efficiency and patient care. Most respondents (74.9%) found the PDA‐based system faster than manual methods for sample labelling and transfusion checks. Satisfaction increased over time, emphasizing the importance of familiarity in digital system integration, consistent with healthcare technology adoption literature [14, 15].

Ongoing health economic evaluation [16], combined with survey data, will provide a comprehensive assessment of the bedside electronic transfusion system's clinical and operational value.

This study's main strength lies in addressing a significant gap by providing large‐scale evidence on staff satisfaction with a BETC system. While previous research has emphasized the safety and benefits of electronic transfusion systems [5, 6, 28], few have explored frontline user experience.

Limitations include a response rate decline from 55% post training to 13% at 6 months, likely due to workload pressures and lack of face‐to‐face facilitation. However, consistent demographics and a substantial second survey sample (*n* = 514) support generalizability. Additionally, self‐selection bias may have influenced responses [31, 32], with individuals holding strong views more likely to participate. Despite these, the positive staff perceptions regarding efficiency and patient safety strongly support broader adoption of electronic transfusion checks.

Conducted at a single NHS Trust, further research should assess generalizability across settings. This study adds a crucial user‐centred perspective to existing safety‐focused evidence. By showing clinical staff not only accept but value BETC integration, it bolsters the case for wider NHS rollout. Future research should examine cost effectiveness, long‐term outcomes and staff engagement across roles to optimize digital transfusion system integration.

In conclusion, the introduction of BETC was associated with high clinical staff satisfaction and perceived benefits in ease of use, time efficiency, patient care, staffing requirements, accuracy and traceability. Satisfaction improved significantly with continued use, indicating strong acceptance of the system over time. These results provide evidence supporting BETC as a favourable alternative to manual bedside checks, with potential to enhance transfusion safety, efficiency and the overall staff experience.

## CONFLICT OF INTEREST STATEMENT

The authors declare no conflicts of interest.

## Data Availability

The data that support the findings of this study are available on request from the corresponding author. The data are not publicly available due to privacy or ethical restrictions.

## APPENDIX A SURVEY QUESTIONS

**Table:**

	Fair	Poor	Good	Very good	Excellent
Ease of use					
Accuracy of performing steps					

1. What is the date for this training? Please input date (dd/mm/yyyy)
2. What is your job role?
3. Which hospital are you working at? Select your answer
  - Hospital 1
  - Hospital 2
  - Hospital 3
4. Please state your specialty (e.g., medicine, surgery, maternity etc.)
5. Which ward are you working on?
6. Your years of experience in this job?
  - <2 years
  - 2–3 years
  - 3–4 years
  - 4–5 years
  - 5+ years
7. How did you receive today's training?
8. Is today's training a one‐off training or a top‐up training session?
9. How long was today's training session?
10. How satisfied are you with the training received to carry out transfusion procedures using the personal digital assistant (PDA)?
11. Do you feel you may require additional training?
12. Are there any barriers you experienced whilst using the PDA?
13. Do you have any additional feedback or suggestions you would like to share to improve the service?
14. Are you trained to perform blood sample collection?
  - Yes
  - No
15. Compared to current manual labelling of samples for blood transfusion, how would you rate the use of PDA for blood sample collection?

**Table:**

	Very slow	Slow	No difference	Fast	Very fast
Current manual method					
Personal digital assistant (PDA)					

16 How would you rate the speed (or efficiency) of labelling transfusion samples:

**Table:**

	Strongly disagree	Disagree	Neither agree nor disagree	Agree	Strongly agree
Patient's care					
Healthcare professional's time					
Number of people required to perform checks					
Traceability of blood components					

17 Do you think the use of the PDAs will reduce the rejection rates for miss‐labelled samples?
  - Yes
  - No
18 Are you trained to perform blood administration?
  - Yes
  - No
19 Compared to the manual system, do you think using the PDA for pre‐transfusion checks is an improvement to

**Table:**

	Fair	Poor	Good	Very good	Excellent
Ease of use					
Accuracy of performing steps					

20 Compared to current manual processes for pre‐transfusion checks, how would you rate the use of PDA for pre‐transfusion checks?

**Table:**

0	1	2	3	4	5	6	7	8	9	10
Very dissatisfied							Very satisfied			

21 Overall, how satisfied are you with using the PDA for pre‐transfusion checks?
